# DIFFERENCES IN CAREGIVING BURDEN ACROSS THE INTERSECTIONALITY OF RACE AND GENDER

**DOI:** 10.1093/geroni/igad104.0801

**Published:** 2023-12-21

**Authors:** Ruotong Mona Liu, Iris Chi, Shinyi Wu

**Affiliations:** Oregon Health & Science University, Portland, Oregon, United States; USC, Los Angeles, California, United States; University of Southern California, Los Angeles, California, United States

## Abstract

Caregivers may be at different risks of various types of burdens by virtue of their gender and racial/ethnic status. This study explored the differences in caregiving burdens among caregivers to people with dementia across the intersectionality of race and gender. Using Round 5 (conducted in 2015) and Round 7 (conducted in 2017) of the National Study of Caregiving and National Health and Aging Trends Study data, the study examined differences in caregiver burdens across and within different gender and racial/ethnic groups, within the realms of financial, emotional, and physical burdens. The sample consisted of 1,206 caregivers who provided services to Medicare beneficiaries. Logistic regressions were performed to assess the three types of burdens each subgroup was experiencing. Results indicated that within the intersectionality framework, compared to White female caregivers, Black male caregivers were 3.3 times (95% confidence interval [CI] 1.77-6.22) more likely to experience financial burden, and Black female caregivers were 54% (95% CI 0.28-0.76) less likely to experience physical burden. Surprisingly, compared to White female caregivers, all the other groups were 37% (95% CI 0.41-0.95) to 71% (95% CI 0.15-0.56) less likely to have emotional burden. The findings highlighted that Black male caregivers are experiencing financial burden and White female caregivers are experiencing emotional burden disproportionately. To develop effective interventions and programs for dementia caregivers, a special focus should be put on monitoring the differences in the types of burdens that the above-mentioned population subgroups experience.

